# Biogeographical Interpretation of Elevational Patterns of Genus Diversity of Seed Plants in Nepal

**DOI:** 10.1371/journal.pone.0140992

**Published:** 2015-10-21

**Authors:** Miao Li, Jianmeng Feng

**Affiliations:** Department of Life Science and Chemistry, Dali University, Dali, China; Wuhan Botanical Garden,CAS, CHINA

## Abstract

This study tests if the biogeographical affinities of genera are relevant for explaining elevational plant diversity patterns in Nepal. We used simultaneous autoregressive (SAR) models to investigate the explanatory power of several predictors in explaining the diversity-elevation relationships shown in genera with different biogeographical affinities. Delta akaike information criterion (ΔAIC) was used for multi-model inferences and selections. Our results showed that both the total and tropical genus diversity peaked below the mid-point of the elevational gradient, whereas that of temperate genera had a nearly symmetrical, unimodal relationship with elevation. The proportion of temperate genera increased markedly with elevation, while that of tropical genera declined. Compared to tropical genera, temperate genera had wider elevational ranges and were observed at higher elevations. Water-related variables, rather than mid-domain effects (MDE), were the most significant predictors of elevational patterns of tropical genus diversity. The temperate genus diversity was influenced by energy availability, but only in quadratic terms of the models. Though climatic factors and mid-domain effects jointly explained most of the variation in the diversity of temperate genera with elevation, the former played stronger roles. Total genus diversity was most strongly influenced by climate and the floristic overlap of tropical and temperate floras, while the influences of mid-domain effects were relatively weak. The influences of water-related and energy-related variables may vary with biogeographical affinities. The elevational patterns may be most closely related to climatic factors, while MDE may somewhat modify the patterns. Caution is needed when investigating the causal factors underlying diversity patterns for large taxonomic groups composed of taxa of different biogeographical affinities. Right-skewed diversity-elevation patterns may be produced by the differential response of taxa with varying biogeographical affinities to climatic factors and MDE.

## Introduction

Biodiversity is unevenly distributed across the globe. Understanding what mechanisms generate geographical variation in biodiversity is one of the fundamental goals of biogeography and macroecology [1]. Though knowledge of elevational diversity patterns has been accumulating in recent decades, the underlying mechanisms remain in controversy. Two frequently observed large-scale elevational patterns in biodiversity exist: hump-shaped diversity-elevation curves and a decrease in diversity with elevation [2,3]. Although species diversity-energy availability hypothesis has been frequently invoked to explain elevational diversity patterns, controversy remains as to the nature of the relationship (linear or curvilinear) and the potential underlying mechanisms [4,5]. For example, Ding *et al*. (2005) observed that when energy availability exceeded a certain level, bird species diversity decreased in Taiwan, resulting in a hump-shaped relationship between energy availability and species diversity [6]. However, Beck and Chey (2008) observed that declining temperature was the best explanation for the decreasing diversity of geometrid moths towards higher elevations in Borneo, i.e. there was a positive, linear relationship between temperature and geometrid moth diversity [7]. Interestingly, Wang *et al*. (2011) noted that the explanatory power of mean temperature in the coldest quarter for latitudinal species diversity may depend on the biogeographical affinities of the studied species [8]. Lomolino (2001) proposed a framework for future studies on elevational diversity patterns, and suggested that the influences of environmental variables may be associated with biogeographical variation [9]. We thus aimed to test a hypothesis that biogeographical affinity may modify the effects of energy availability on elevational taxonomic diversity.

One of the most pervasive explanations for hump-shaped diversity patterns is the mid-domain effects (MDE). MDE suggests that the diversity peaks in mid-elevation are caused by the increasing overlap of species ranges towards the center of the domain or elevational gradient [10,11]. However, the influence and generality of such geometric constraint models remain in debate [12–15]. The influences of MDE may vary with the biogeographical origins of the studied taxa; for example, compared to tropical taxa, the diversity of temperate taxa over an elevational gradient may be more strongly associated with MDE [16,17]. MDE are seemed to be stronger for broad-ranged taxa than for narrow-ranged ones, and temperate species generally occur over wider elevational ranges, as predicted by Rapoport’s rule [18]. Hence, this may explain the differential importance of MDE on the diversity of temperate versus tropical taxa.

Nepal harbors the longest elevational gradient on the Earth. Within a relatively short horizontal distance, the land traverses large climatic and environmental gradients, from subtropical forests in the lowlands, to glaciated, snowbound upper peaks [19]. Thus, Nepal is an ideal place for testing a variety of biogeographical hypotheses, especially those related to diversity-elevation patterns [20,21]. The diversity of various taxonomic groups has been examined over elevational gradients in Nepal, including that of birds and mammals [22], ferns [23], flowering plants [21,24–26], lichens [27], liverworts and mosses [28], and orchids [29]. Two key findings are that factors causing variation in taxonomic diversity may differ among organisms, and that elevational patterns may be taxonomic group specific [26, 28]. This suggests that diversity-elevation relationship may depend on the interactions between eco-physiological traits of specific taxa and climatic or environmental factors the taxa experienced along the gradient. As species or genus’s eco-physiological traits may be strongly related to its biogeographical affinity [30], we therefore hypothesized that taxa of different biogeographical affinities may show different elevational patterns of diversity [16,17]. However, few relevant studies have been conducted for Nepal. Therefore, one of the major aims of our study was to compare elevational patterns of plant diversity in Nepal in terms of taxa’ biogeographical affinities.

Grytnes & Vetaas (2002) proposed that the diversity peak in mid-elevation may be due to the intermediate location between the ranges of temperate and tropical flora, which increases the chance of immigration from both directions, i.e. the mass effect [24]. That is, tropical floras may immigrate from lower elevations, whereas temperate floras may immigrate from higher elevations, resulting in an overlap of tropical and temperate floras at mid-elevations and a resultant hump-shaped diversity-elevation pattern. To test this hypothesis, we constructed an index of floristic overlap to investigate the role of floristic overlap on elevational patterns of genus diversity.

In the present study, we tested the following hypotheses: (i) plants with different biogeographical affinities show different elevational diversity patterns; (ii) floristic overlap strongly shapes the elevational patterns observed in the total diversity of genera; and, (iii) biogeographical affinity modifies the influence of mid-domain effects and climatic factors.

## Materials and Methods

### Study area description

Our study area encompasses the entire Nepal, which covers 900 km from east to west on the southern slopes of the Central Himalayas (80°04'–88°12' E, 26°22'–30°27' N). Three south-eastern to north-western mountain ranges form the main body of Nepal, including the Siwalik range (maximum elevation of 1,000–1,500 m), the Mahabharat range (2,700–3,000 m) and the Great Himalayas (5,000–8,000 m) [31, 32]. Topographically, the study area has the longest elevational gradient in the world, extending from 60 m a.s.l. to more than 8,000 m a.s.l. over a distance of 150–200 km, and running from the south to the north [31–33].

Nepal harbors a wide range of climatic conditions, which vary with seasons and geography. Overall, annual precipitation ranges from 1,500 to over 3,000 mm [33]. On the basis of seasonal variation in precipitation, climate in Nepal can be classified into a dry winter period and a wet summer period, with the majority of rainfall occurring from mid-June to mid-September [19]. Precipitation increases gradually from west to east, and hence the eastern part of Nepal is comparatively wetter [19, 34]. Annual precipitation initially increases with elevation until up to 2,000 m a.s.l. in Northern Nepal, and then decreases with the increase of elevation [32,34]. At higher elevations, precipitation occurs in the form of snow [19, 34].

The mean annual temperature at 100 m a.s.l. in Nepal is around 24.7°C, and with increasing elevation, energy availability steeply decreases and vegetation types range widely [19,26]. Dominant vegetation types on elevation are as follows: (1) mixed evergreen and drought-deciduous monsoon forest below c. 1000 m; going from 1,000 to 2,000 m a.s.l., tropical/subtropical vegetation is gradually replaced by warm-temperate forest; between 2,000–3,000 m a.s.l., temperate vegetation dominated by oak and laurel forests is the dominant vegetation type; the sub-alpine zone (3000–4000 m) is dominated by *Betula utilis* and coniferous forest., while the upper elevational limits for *Abies spectabilis* and *Pinus wallichiana* forest are 4,000–4,300 m a.s.l; in the alpine zone above 4,000 m, grasslands gain dominance, while in the high alpine zone genera such as *Saxifraga*, *Gentiana* and *Androsace* become common [19,35].

### Plant data sources

Plant species data and nomenclature used in the present study were taken from the online version of the Annotated Checklist of the Flowering Plants of Nepal (http://www.efloras.org/, accessed on Dec 1^st^ in 2013) (S1 Table). The information provided by this database included species identity, genus name, and family for each species as well as elevational range. A few cases of varieties and subspecies were considered as separate taxa in this database. In the present study, genus diversity instead of species diversity was used to describe plant diversity over the elevational gradient in order to reduce the possibility of sampling bias, as higher taxonomic levels are less likely to be missed in surveys [36,37].

### Subdivision of the study area into elevational bands

We focused on elevational diversity patterns between 60 and 6,000 m a.s.l., as suggested by Grytnes & Vetaas (2002) [24]. The study area was divided by 100-m vertical interval and resulted in 60 elevational bands.

### Source data for explanatory variables

**Fig 1:**
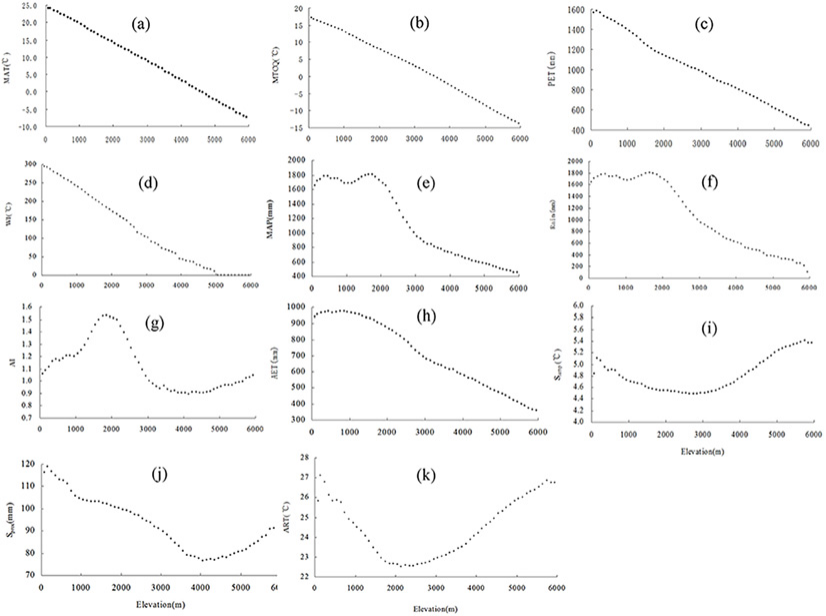
Climatic factors on elevation. Subgraphs: (a) = mean annual temperature (MAT), (b) = mean temperature of the coldest quarter (MTCQ), (c) = annual potential evapotranspiration (PET), (d) = warmth index (WI), (e) = mean annual precipitation (MAP), (f) = Rain, (g) = aridity index (AI), (h) = annual actual evapotranspiration (AET), (i) = temperature seasonality (S_Temp_), (j) = precipitation seasonality (S_Prec_), and (k) = annual range in temperature (ART).

We examined the relationships between genus diversity and thirteen potential explanatory variables as follows: (1) the index of floristic overlap (IFO; the relative proportion of both tropical and temperate genera), (2) mean annual temperature (MAT, °C), (3) mean temperature of the coldest quarter (MTCQ, °C), (4) annual potential evapotranspiration (PET, mm), (5) warmth index (WI, °C; the sum of mean monthly temperatures when > 5°C), (6) mean annual precipitation (MAP, mm), (7) rainfall (mm; the sum of the mean monthly precipitation when mean monthly temperature > 0°C), (8) aridity index (AI; mean annual precipitation divided by annual actual evapotranspiration), (9) annual actual evapotranspiration (AET, mm), (10) temperature seasonality (S_Temp_, °C; the standard deviation of mean monthly temperature), (11) precipitation seasonality (S_Prec_, mm; coefficient of variation of mean monthly precipitation), (12) the annual range in temperature (ART, °C; maximum temperature of the warmest month–the minimum temperature of the coldest month), and (13) mid-domain effects (MDE). These explanatory variables, with the exception of IFO and mid-domain effects, were grouped into four categories (Table 1): energy availability, water availability, energy-water balance, and seasonality. All source data for species distribution areas, MAT, MTCQ, MAP, S_Temp_, S_Prec_, ART, and mean monthly precipitation and temperature (needed for estimating rainfall and WI) were taken from WorldClim-Global Climate Data with a 30 second resolution [38] (http://www.worldclim.org/, accessed on Jul 1^st^ 2014). PET, AET, and AI with a 30 second resolution were downloaded from the Consortium for Spatial Information (CGIAR-CSI) [39,40] (http://www.cgiar-csi.org/, accessed on Jul 1^st^ 2014). All of the climatic factors on elevation can be found in Fig 1.

**Table 1:** Coefficients of determination (*R* ^*2*^) for each predictor in simultaneous autoregressive regressions in linear and quadratic forms.

Predictor	Tropical genus diversity“^L^”	Temperate genus diversity“^L^”	Total genus diversity“^L^”	Tropical genus diversity“^Q^”	Temperate genus diversity“^Q^”	Total genus diversity“^Q^”
**IFO**			0.382***			0.641***
**MDE**	<0.001	0.87***	0.169**	0.035	0.899***	0.154**
**Energy availability**						
MAT	0.601***	0.006	0.468***	0.337***	0.920***	0.549***
MTCQ	0.600***	0.011	0.487***	0.209**	0.895***	0.491***
PET	0.584***	<0.001	0.402***	0.432***	0.907***	0.640***
WI	0.708***	0.003	0.446***	0.733***	0.885***	0.873***
**Water availability**						
MAP	0.841***	<0.001	0.615***	0.807***	0.521***	0.632***
RAIN	0.810***	0.004	0.644***	0.777***	0.499***	0.565***
AI	0.552***	0.016	0.477***	0.704***	0.034	0.517***
**Energy-water balance**						
AET	0.788***	0.001	0.622***	0.655***	0.755***	0.482***
**Seasonality**						
S_Temp_	0.033	0.700***	0.523***	0.047	0.756***	0.502***
S_Prec_	0.611***	0.043	0.238***	0.663***	0.137*	0.369***
ART	<0.001	0.820***	0.428***	<0.001	0.814***	0.418***

“*” = *P*<0.05

“**” = *P*<0.01

“***” = *P*<0.001.

“^L^”, linear predictor

“^Q^”, quadratic predictor.

IFO, the index of floristic overlap; MDE, mid-domain effects; MAT, mean annual temperature; MTCQ, mean temperature of the coldest quarter; PET, annual potential evapotranspiration; WI, warmth index; MAP, mean annual precipitation; Rain, rainfall; AI, aridity index; AET, annual actual evapotranspiration; S_temp_, temperature seasonality; S_Prec_, precipitation seasonality; ART, annual range in temperature.

Several analytical and simulative models had been proposed to evaluate the role of MDE [10,11]. We used the Mid-Domain Null Program to generate null diversity patterns as predicted by MDE [41]. We simulated genus diversity over the elevational gradient for all of the studied genera, and for genera belonging to different biogeographical affinities separately, using observed range sizes without replacement and randomly chosen range midpoints to produce ranges within the domain limits (S2 Table). This approach eliminates any bias caused by the differences between theoretical frequency distributions of range sizes and the observed ones [11,17,41]. We built a python model in ArcGIS Desktop10.2^TM^ to obtain the data of other explanatory variables (with the exception of IFO and MDE) over the elevational gradient (Fig 1). In this python model, we obtained the data of all explanatory variables for each grid on all elevational bands, and then obtained their average values.

The area for each elevational band was calculated with the Digital Elevation Model data (DEM) obtained from WorldClim-Global Climate Data with a 30 second resolution [38] (S3 Table). The total area of each elevational band was the product of grid number times grid area. Following the methodology proposed by previous studies [36,37,42], we used genus diversity per unit area, as *genus diversity* = *genus richness* / ln(*area*), to represent plant diversity, in order to account for the effects of area.

### Interpolation of elevational presences of genera

We interpolated the presence of each genus on the basis of recorded altitudinal range of each genus (between maximum and minimum altitudes). This method assumed that taxa are continuously distributed between their upper and lower limits (i.e. present in every 100-m vertical band) [24,43]. Although this may create an artificially humped pattern [24], one of our major goals was to compare elevation-genus diversity patterns for genera of different biogeographical affinities, and as all genera were treated in the same way, any differences among genera are therefore unlikely to be an artifact of this interpolation method [28]. Through this interpolation, we found 1403 genera that occurred from 60 to 6,000 m a.s.l. (S4 Table).

### Biogeographical affinities of genera

We used the system proposed by Wu (1991) [44] for the classification of biogeographical affinities at genus level to assign biogeographical affinities to seed plant genera in Nepal. This system is arguably one of the most important ones for flora classification worldwide [36,45–47]. In this large scale classification system, the biogeographical affinity of a given genus is primarily defined or determined on the basis of its biogeographical history, fossil records, and especially modern distribution centers. For example, if a given genus shows its distribution centers are in tropical regions, this system groups it into tropical affinities. In the present study, we grouped genera into three types of biogeographical affinities: tropical, temperate and cosmopolitan. We determined the biogeographical affinities for 1323 genera, or 94.3% of the total, including 724 tropical genera, 519 temperate genera and 80 cosmopolitan genera (S4 Table). As tropical and temperate genera comprised 88.6% of the overall genera and the proportion of cosmopolitan genera was very low (5.7%), only temperate and tropical genera were considered in this study.

### Elevational patterns of genus diversity

To investigate elevational patterns in genus diversity, the numbers of tropical, temperate and total genera in each elevational band were tallied. To further explore the elevational diversity patterns of genera with different biogeographical affinities, the proportions of tropical and temperate genera in each elevational band were calculated. All datasets followed normal distributions, which is an important precondition for regression analysis.

### Index of floristic overlap over the elevational gradient

As floristic overlap, i.e., the co-occurrence of tropical and temperate flora, may be associated with elevational patterns of taxonomic diversity [24,48], we constructed an index of floristic overlap. It was used to reflect the relative proportions of the two biogeographical groups, and was calculated as the ratio of the number of tropical to temperate genera in each elevational band [48]. When the number of tropical genera in each elevational band equaled the number of temperate ones, the strength of floristic overlap reached its maximum with respect to our index (excluding the cases when the total number of tropical and temperate genera was zero), whereas when only tropical or temperate genera were observed in an elevational band the index strength was zero, i.e. no floristic overlap was observed. In addition, when the index was more than 1, we adopted its reciprocal, i.e., the ratio of the number of temperate to tropical genera in each elevational band. In other words, when the index equaled 1, floristic overlap was maximized. Scatter diagrams were plotted to illustrate the patterns of floristic overlap index over the elevational gradient.

### Predictors of elevational patterns in genus diversity

Spatial autocorrelation is frequently observed in elevational taxonomic diversity data, which may result in pseudo-significance of the results as the type I error rate is inflated in significance tests [49]. Besides, the estimates of model coefficients may also be influenced by spatial autocorrelation in model residuals [50,51]. To account for the effects of spatial autocorrelation, simultaneous autoregressive (SAR) models were used to investigate the explanatory power of each predictor and the models′ significance [52]. In order to find the best-fit model with the lowest possible AIC (Akaike information criterion), which rewards goodness of fit (as assessed by the likelihood function) and penalizes models with greater complexity [53,54], we used SAM 4.0 (www.ecoevol.ufg.br/sam/) to generate SAR models of all possible combinations of the aforementioned predictors [52,55] (S5 Table). We used the following methodologies to select the candidate predictors: (1) the best predictor must be included in all of the models. (2) to avoid multicollinearity, only the best predictor in each variable group in SAR models can be the candidate predictor. (3) only the predictor showed significant roles in SAR models was allowed to enter the model. To account for the effects of spatial autocorrelation in model selection, corrected AICs of all possible SAR models were used to evaluate model performance. Following Burnham & Anderson (2002) [53], the best-fit model had the lowest corrected AIC and any models with a ΔAIC of less than two in comparison with the best model were considered an equally good fit to the data. In addition to the linear term, we also included a quadratic term for each predictor in the SAR models to allow for nonlinear relationships. To investigate the relative influences of MDE and climatic factors on temperate genus richness, we conducted a partial regression analysis with SAM 4.0 (www.ecoevol.ufg.br/sam/) [52,55].

## Results

### Elevational patterns of genus diversity

**Fig 2:**
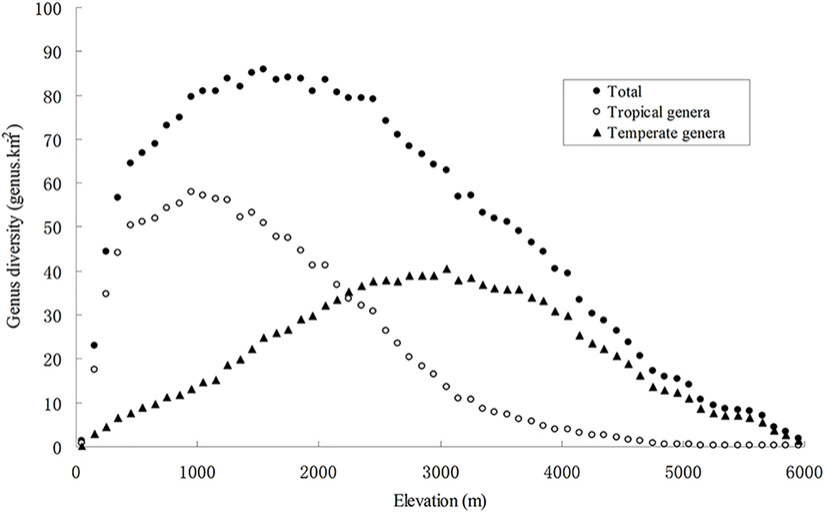
The relationship between genus diversity and elevation.

Tropical, temperate and total genus diversity showed hump-shaped distributions along the elevational gradient (Fig 2). Maximum tropical and total genus diversity were observed around 900–1,100 and 1,500–1,700 m a.s.l., respectively, and both had right-skewed unimodal distributions, whereas the maximum temperate genus diversity occurred around 3,000–3,100 m a.s.l. with a nearly-symmetrical unimodal distribution (Fig 2).

### Floristic overlap and proportions of different biogeographical groups along the elevational gradient

**Fig 3:**
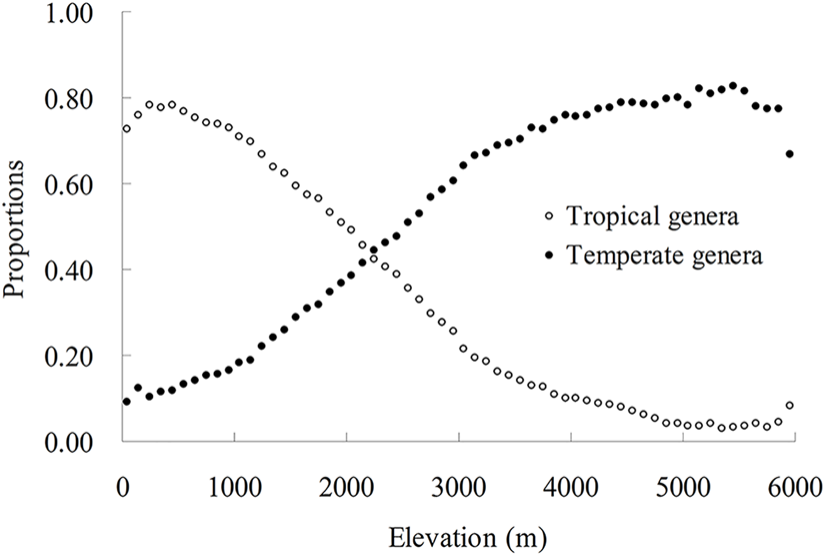
The proportion of tropical and temperate genera along the elevation gradients.

**Fig 4:**
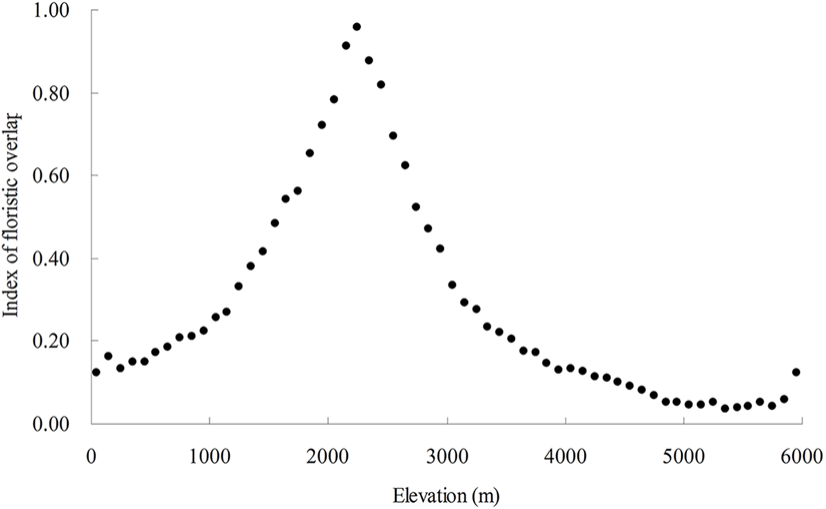
The index of floristic overlap with elevation.

The proportion of temperate genera increased with elevation (*P*<0.05), while tropical genera showed the opposite pattern (*P*<0.05) (Fig 3). The floristic overlap index had a unimodal distribution with elevation, and its maximum was around 2,200–2,300 m a.s.l. (Fig 4).

### Altitudinal ranges and biogeographical affinity

One-way ANOVAs revealed that, compared to tropical genera, temperate genera had wider elevational ranges and were observed at higher elevations (p< 0.001 for both).

### Interpretation of elevational patterns of genus diversity

**Fig 5:**
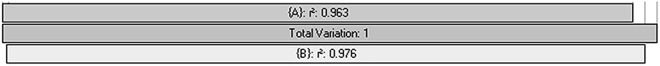
Comparing the effects of climatic and the mid-domain effects on temperate genus diversity by partial regression. A shows the mid-domain effects; B shows climatic effects. Total variance explained by {A} = 0.963; Total variance explained by {B} = 0.976; Total variance explained by {A+B} = 0.996. [A.B] variance explained by {A} only = 0.019; [A:B] Variance Sharely explained = 0.944; [B.A] Variance explained by {B} only = 0.032; [1-(A+B)] Unexplained variance = 0.004. Moran′s index of residuals in the model was 0.017 at first class.

No significant influences of MDE were detected on the elevational variation in tropical genus diversity (*P*>0.05), either in linear or quadratic simultaneous autoregressive (SAR) models, whereas its distribution was significantly influenced by most of the tested climatic factors, and the strongest predictor was MAP in linear form (Table 1). In contrast to tropical genera, MDE accounted for most of the elevational variation in temperate genus diversity (Table 1). Also, for temperate genera, the categories of energy availability, water availability and energy-water balance in quadratic terms significantly affected elevational variation in diversity with energy availability explaining most of the variation, and the strongest predictors was MAT in quadratic form (Table 1). Climatic factors and the index of floristic overlap significantly affected the relationship between elevation and total genus diversity (Table 1). In model inference and selection, MAP, AET, WI, WI^2^, S_prec,_and S_prec_^2^ were included as candidate predictors for tropical genus diversity; for temperate genus diversity, predictors were MDE, MDE^2^, MAT, MAT^2^, MAP, MAP^2^, AET, AET^2^ and ART; and for total genus diversity, predictors were IFO, IFO^2^, MDE, RAIN, AET, Stemp, WI and WI^2^ (Table 2); and there were 8, 16 and 32 possible SAR models, respectively (also see S5 Table). The best-fit model for tropical genus diversity included S_temp_, S_temp_^2^, and MAP as its predictors, while for temperate genus diversity the best model comprised MAP, MAP^2^, ART, MAT and MAT^2^, and for total diversity, IFO, IFO^2^, AET, Stemp, WI, and WI^2^ entered into the best model (Table 2). The partial regression analysis showed that climatic factors and MDE individually explained 3.4% and 1.9% of the elevational variation in temperate genus diversity, respectively, and jointly explained 94.4% of the variation (Fig 5).

**Table 2:** Coefficients of determination (*R* ^*2*^) and Akaike information criterions (AIC) of the best SAR models. There were 8, 16 and 32 possible SAR models for tropical, temperate and total genus diversity, respectively (see S5 Table). For each biogeographical group, the ΔAICc compares the best model (ΔAICc = 0) with all of models generated, and any models with a ΔAICc of less than two in comparison with the best model were considered an equally good fit to the data.

Responses	Predictors	nVars	*R* ^*2*^	AICc	ΔAIC_c_
Tropical genus diversity	S_temp_, S_temp_^2^, MAP	3	0.867	431.23	0.00
	AET, S_temp_, S_temp_^2^, MAP	4	0.869	432.93	1.70
Temperate genus diversity	MAP, MAP^2^, ART, MAT, MAT^2^	5	0.960	301.39	0.00
Total genus diversity	IFO, IFO^2^, AET, Stemp, WI, WI^2^	6	0.968	388.27	0.00

## Discussion

### Proportions of genera with different biogeographical affinities over the elevational gradient

Wang *et al*. (2011) observed that the proportions of tropical versus temperate species showed the opposite trends over a latitudinal gradient: the proportion of tropical species decreased with latitude, while temperate species increased [8]. Taking elevational gradient as a surrogate for latitude, we observed identical contrasting trends for tropical and temperate genera in this study. These observations may suggest that biogeographical affinities reflect different tolerances of energy insufficiency [56–58]. Most taxa of tropical affinities are weak tolerant of energy insufficiency, and hence can be easily excluded by the decreasing availability of energy towards higher elevations or latitudes, and by contrast, temperate taxa may have stronger tolerance of energy insufficiency [8].

### Effects of energy variables and biogeographical affinity

The linear regression analyses in this study showed that most of energy related variables strongly and linearly influenced elevational patterns of tropical genus diversity. This suggests that the eco-physiological traits of tropical genera, as indicated by their tropical biogeographical affinities, may be best suited to the warmer environments at lower elevations, and the decreasing thermal energy with elevation may filter out tropical genera. Our findings, therefore, strongly support the species-energy hypothesis (or tropical conservatism hypothesis) [56–58].

In contrast, we did not observe the negative (linear) relationship between temperate genus diversity and energy variables, all of which decreased linearly with elevation. Oommen & Shanker (2005) found that temperate species diversity showed a nonlinear relationship with temperature [16]. Partially consistent with their findings, we found that energy variables explained most of the elevational variation in diversity (in the quadratic terms of the regressions). This result implies that the eco-physiological traits of temperate genera, as indicated by their temperate biogeographical affinities, may be best suited to relatively cool environments, and are constrained by extremely harsh climates (such as very cold temperatures) at high elevation regions and by warmer climates at low elevation regions. This would result in lower diversity of temperate genera at both low and high elevations and produce a nearly-symmetrical unimodal diversity pattern. McCain (2007) proposed a climatic model where favorable climate conditions (e.g. of temperature and precipitation) around mid-elevations contribute to the hump-shaped diversity patterns [59]. Our observation is consistent with this climatic model for temperate genera.

In summary, the different responses of temperate and tropical genera to energy availability may be one of the important reasons for their differences in elevational diversity patterns. To a certain extent, this confirms one of our main hypotheses that biogeographical affinity may interact with elevation to produce different patterns of taxonomic diversity via modulating the effects of climatic factors.

Both Wang *et al*. (2007) and Oommen & Shanker (2005) suggested that, compared to temperate genera, tropical genus diversity is more strongly influenced by climatic factors [16,17]. In contrast, in the present study, the influence of climatic factors on temperate genus diversity was not weaker than it was on tropical genus diversity. This contrast may be due to different analytical methods; for example, previous studies used only linear regressions to explore the predictive value of climatic factors, whereas we also included quadratic terms for a better fit.

### Influences of water and energy variables and biogeographical affinities

Hawkins et al. (2003) proposed that water variables usually showed the strongest influences on taxonomic diversity in low latitudes, whereas energy variables or water–energy variables represented the strongest predictors in high latitudes, as in low latitude regions the energy input is abundant and in high latitude regions the energy input is low, suggesting that the relative effects of water variables and energy or water-energy variables may depend on latitude [60]. Studies by Bhattarai et al. (2004) and Kessler et al. (2011) lent support to this proposal on elevation, i.e., energy-related variables probably controlled fern species diversity directly at higher elevations but at lower elevations the effect was more probably related to moisture [23, 61]. Our observations showed that for tropical genus diversity, the strongest predictor was MAP, whereas for temperate genus diversity, it was MAT. Most of tropical genera in the present study were observed in lower elevations where energy is abundant, and thus the influence of energy related variables may be weaker than those of water related variables. However, temperate genera may be best suited to relatively cool environments, being constrained by extremely harsh climates (such as extremely energy deficiency) at high elevations and by warmer climates at lower elevations, and hence were mostly observed in mid-elevations, resulting in energy variables playing strong roles in quadratic terms. In close, the relative importance of water related and energy related variables may not only depend on latitude and elevation, but also vary with biogeographical affinities.

### Mid-domain effects and biogeographical affinity

Mid-domain effects did not explain the diversity-elevation relationship of tropical genera, but they strongly influenced temperate genus diversity, consistent with the findings of Oommen & Shanker (2005) [16] and Wang *et al*. (2007) [17]. The inconsistent response of tropical and temperate genera could be explained by the narrower tolerances of tropical taxa to environmental variation and hence narrower elevational ranges predicted by Rapoport’s rules was confirmed in the present study. Hence, the influence of MDE would be reduced in tropical taxa, as their narrower elevational ranges would overlap to a lesser degree towards the middle of the gradient [16]. In addition, the tropical taxa are more likely to be influenced by the steep temperature gradient than by MDE, as our results showed. MDE may therefore be over-shadowed by the effects of the climatic gradient, especially by the energy availability, resulting in an overall decrease in tropical genus diversity with elevation [16].

Our study did not observe consistent influence of MDE, i.e., the influence of MDE varied with biogeographical affinities, and for total genus diversity, the influence of MDE was weaker than that of climatic factors (Table 1). Thus, a noteworthy and strong contribution of MDE was only for temperate genus richness, which, however, should not be overemphasized because of stronger influence of climatic factors (Table 1) and statistical challenges of adequately disentangling their relevant effects (Fig 5). By contrast, we observed consistent roles of climatic factors, i.e., climatic factors showed the strongest influences on tropical, temperate and total genus diversity. We thus suggested that MDE may not be the main driving factor of the elevational patterns of genus diversity of seed plants in Nepal, although its influences may certainly not be ruled out, which was supported by a variety of previous studies [14,62–64]. In sum, the elevational patterns may be most closely related to climatic factors, while MDE may modify the patterns to some extent, which was consistent with the previous studies conducted in Nepal [21, 28].

### Elevational patterns of total genus diversity

Genera in the present study were largely either of tropical or temperate affinities, and different biogeographical groups showed different elevational diversity patterns as a result of different influences of MDE and climatic factors. These results suggest that elevational patterns in total genus diversity, even within similar elevational gradients with similar biogeographical history, cannot be explained by the same way [65]. Multiple mechanisms likely drive elevational diversity patterns, and caution is needed when summarizing the controlling factors for large taxonomic groups (e.g. the seed plants) which include species of different biogeographical affinities.

### Right-skewed distributions of total genus diversity with elevation

A variety of factors or hypotheses have been proposed to explain the unimodal relationship between diversity and elevation, such as water-energy dynamics [26], area [9,17] and spatial constraints (e.g., the mid-domain effect) [10,11]. However, Guo *et al*. (2013) argued that most of these putative explanations fail to explain why many distributions are right-skewed (maximum diversity below the middle of the gradient), with the exception of one hypothesis that spatial constraints reduce diversification and immigration at higher elevations [66], thereby increasing the risk of extirpation/extinction [66,67]. However, our study cannot comment on this hypothesis. Grytnes & Vetaas (2002) speculated that the right-skewed patterns observed in Nepal were caused by an underlying linear decrease in diversity with elevation, in combination with hard boundaries and/or interpolation [24]. Here, we found the pattern may be a combination of a unimodal distribution for tropical genus diversity that peaks at low elevations and a nearly-symmetrical unimodal distribution for temperate genus diversity. Hence, right-skewed distributions of total genus diversity with elevation may be driven by the contrasting influences of MDE on tropical and temperate genera and the differential responses of tropical and temperate genera to climatic factors on the elevational gradient.

### Floristic overlap and elevational diversity patterns

Bhattarai & Vetaas (2003) suggested that the mid-elevation peak in plant diversity in Nepal may be linked to its intermediate location between the ranges of temperate Himalayan flora at high elevations and of subtropical flora at low elevations [26]. Our study provides quantitative support for this hypothesis. The index of floristic overlap, which reflects the magnitude of co-occurrence of temperate and tropical floras, showed a unimodal relationship with elevation and explained a substantial part of the elevational variation in total genus diversity in Nepal. The underlying biological mechanisms involved are probably related to source-sink dynamics [24,68,69]. That is, tropical genera disperse from lower elevations, whereas temperate genera disperse from higher elevation, resulting in unimodal patterns of genus diversity over the elevational gradient.

### Uncertainty and weakness

One of the weaknesses of the present study is the fact that both climatic variables and genus diversity along the elevation gradients were reduced to elevational bands, hence trends are highly smoothed, which, to certain extent, may result in extremely high explanatory power (*R* ^2^ = 0.96) of the multiple models. Another weakness is that we only used Flora of Nepal as data source (lacking of collected data) and used linear interpolation of range extremes of genera, thereby obtaining genus diversity on elevation. Though this method has been widely used in the publications on elevational patterns of the Nepal flora, its weakness can not be denied. Firstly, compared with plot based study, this method can not efficiently minimize the area-effect as suggested by Lomolino (2001) [9]. Secondly, our linear interpolation may add genera at locations where they in fact are not present, thus enhancing artificial geometric effects like MDE [24]. Thirdly, the interpolation method may have ignored disjunctive patterns of genus diversity caused by extinctions provoked by the uplift of this mountain range and longitudinal variation of humidity. Losos (2003) argued that ecological and phylogenetic similarities are often not related to each other, suggesting high probability of disjunctive distribution patterns of genera [70]. Given the uplift of the Himalayan Range provoked extinctions in history, it may also cause disjunctive distribution of genera. For example, Holarctic and paleotropical species of the same genus may be far from overlapping. Besides the energy gradient on elevation, water availability gradient on longitude may also significantly influence geographical patterns of genus diversity. Their combined effects may cause species niches present in low elevation regions in the east and in high elevation regions in the west where thermal requirements may be weaker than humidity needs. Thus, the smoothing and interpolating algorithms adopted here may bias the real elevational patterns of genus diversity of seed plants in Nepal, and the results and conclusions drawn from this study may be tentative and should be taken with caution.

## Supporting Information

S1 Table Plant species data downloaded from the online version of the Annotated Checklist of the Flowering Plants of Nepal. (XLS)

S2 Table Genus richness predicted by MDE. (XLS)

S3 Table Area over elevation. (XLS)

S4 Table List of genera and their biogeographical affinities. (XLS)

S5 Table Model inference for tropical, temperate and total genus diversity. (DOC)
