# Supplementary material for: Biogeographical Interpretation of Elevational Patterns of Genus Diversity of Seed Plants in Nepal
Source: PLoS One. 2015 Oct 21;10(10):e0140992. doi: 10.1371/journal.pone.0140992 (PMC4619261; doi:10.1371/journal.pone.0140992)
Supplement: S5 Table — (DOC) [file pone.0140992.s005.doc]

S6-1 Model inference for tropical genus diversity

| Model | Predictors | nVars | *R^2^* | AICc | ∆AIC_c_ |
| --- | --- | --- | --- | --- | --- |
| Mod #1 | S_temp_, S_temp_^2^, MAP | 3 | 0.867 | 431.23 | 0.00 |
| Mod #2 | AET, S_temp_, S_temp_^2^, MAP | 4 | 0.869 | 432.93 | 1.70 |
| Mod #3 | MAP | 1 | 0.841 | 437.17 | 5.93 |
| Mod #4 | WI, WI^2^, AET, S_temp_, S_temp_^2^, MAP | 6 | 0.868 | 438.74 | 7.51 |
| Mod #5 | AET, MAP | 2 | 0.830 | 443.32 | 12.09 |
| Mod #6 | WI, WI^2^, MAP | 3 | 0.833 | 444.72 | 13.49 |
| Mod #7 | WI, WI^2^, S_temp_, S_temp_^2^, MAP | 5 | 0.844 | 445.97 | 14.74 |
| Mod #8 | WI, WI^2^, AET, MAP | 4 | 0.830 | 448.55 | 17.32 |

S6-2 Model inference for temperate genus diversity

| Model | Predictors | nVars | *R^2^* | AICc | ∆AICc |
| --- | --- | --- | --- | --- | --- |
| Mod #1 | MAP, MAP^2^, ART, MAT, MAT^2^ | 5 | 0.960 | 301.39 | 0.000 |
| Mod #2 | MAP, MAP^2^, MAT, MAT^2^ | 4 | 0.948 | 315.37 | 13.975 |
| Mod #3 | ART, MAT, MAT^2^ | 3 | 0.945 | 315.85 | 14.463 |
| Mod #4 | MAP, MAP2, AET, AET^2^, MAT, MAT^2^ | 6 | 0.951 | 316.90 | 15.508 |
| Mod #5 | AET, AET^2^, ART, MAT, MAT^2^ | 5 | 0.936 | 330.39 | 29.001 |
| Mod #6 | MAP, MAP^2^, AET, AET2, ART, MAT, MAT^2^ | 7 | 0.940 | 331.79 | 30.400 |
| Mod #7 | MAT, MAT^2^ | 2 | 0.920 | 336.05 | 34.659 |
| Mod #8 | AET, AET^2^, MAT, MAT^2^ | 4 | 0.921 | 339.74 | 38.352 |
| Mod #9 | MDE, MDE^2^, MAT, MAT^2^ | 4 | 0.910 | 348.20 | 46.810 |
| Mod #10 | MDE, MDE^2^, AET, AET2, MAT, MAT^2^ | 6 | 0.908 | 354.81 | 53.419 |
| Mod #11 | MDE, MDE^2^, MAP, MAP^2^, MAT, MAT^2^ | 6 | 0.902 | 358.15 | 56.762 |
| Mod #12 | MDE, MDE^2^, MAP, MAP^2^, AET, AET^2^, MAT, MAT^2^ | 8 | 0.912 | 358.24 | 56.845 |
| Mod #13 | MDE, MDE^2^, AET, AET^2^, ART, MAT, MAT^2^ | 7 | 0.895 | 365.73 | 64.335 |
| Mod #14 | MDE, MDE^2^, ART, MAT, MAT^2^ | 5 | 0.874 | 370.87 | 69.474 |
| Mod #15 | MDE, MDE^2^, MAP, MAP^2^, AET, AET^2^, ART, MAT, MAT^2^ | 9 | 0.893 | 372.94 | 71.545 |
| Mod #16 | MDE, MDE^2^, MAP, MAP^2^, ART, MAT, MAT^2^ | 7 | 0.872 | 377.39 | 76.000 |

S6-3 Model inference for total genus diversity

| Model | Predictors | nVars | *R^2^* | AICc | ∆AIC |
| --- | --- | --- | --- | --- | --- |
| Mod# 1 | IFO, IFO^2^, AET, Stemp, WI, WI^2^ | 6 | 0.968 | 388.27 | 0.00 |
| Mod# 2 | IFO, IFO^2^, RAIN, AET, Stemp, WI, WI^2^ | 7 | 0.968 | 391.12 | 2.85 |
| Mod# 3 | RAIN, AET, Stemp, WI, WI^2^ | 5 | 0.955 | 406.04 | 17.77 |
| Mod# 4 | AET, Stemp, WI, WI^2^ | 4 | 0.947 | 413.00 | 24.73 |
| Mod# 5 | IFO, IFO^2^, RAIN, Stemp, WI, WI^2^ | 6 | 0.942 | 424.43 | 36.16 |
| Mod# 6 | AET, WI, WI^2^ | 3 | 0.92 | 435.68 | 47.41 |
| Mod# 7 | RAIN, AET, WI, WI^2^ | 4 | 0.918 | 439.52 | 51.25 |
| Mod# 8 | IFO, IFO^2^, RAIN, AET, WI, WI^2^ | 6 | 0.918 | 444.57 | 56.30 |
| Mod# 9 | IFO, IFO^2^, AET, WI, WI^2^ | 5 | 0.909 | 448.48 | 60.21 |
| Mod# 10 | RAIN, WI, WI^2^ | 3 | 0.898 | 449.67 | 61.40 |
| Mod# 11 | IFO, IFO^2^, Stemp, WI, WI^2^ | 5 | 0.906 | 450.10 | 61.83 |
| Mod# 12 | RAIN, Stemp, WI, WI^2^ | 4 | 0.901 | 450.85 | 62.58 |
| Mod# 13 | Stemp, WI, WI^2^ | 3 | 0.896 | 451.35 | 63.08 |
| Mod# 14 | IFO, IFO^2^, RAIN, WI, WI^2^ | 5 | 0.904 | 451.35 | 63.08 |
| Mod# 15 | WI,WI^2^ | 2 | 0.873 | 460.40 | 72.13 |
| Mod# 16 | IFO, IFO^2^, WI, WI^2^ | 4 | 0.853 | 474.56 | 86.29 |
| Mod# 17 | IFO, IFO^2^, MDE, AET, Stemp, WI, WI^2^ | 7 | 0.832 | 490.82 | 102.55 |
| Mod# 18 | IFO, IFO^2^, MDE, AET, WI, WI^2^ | 6 | 0.787 | 502.08 | 113.81 |
| Mod# 19 | MDE, AET, WI, WI^2^ | 4 | 0.758 | 504.47 | 116.20 |
| Mod# 20 | MDE, AET, Stemp, WI, WI^2^ | 5 | 0.749 | 509.26 | 120.99 |
| Mod# 21 | MDE, RAIN, AET, Stemp, WI, WI^2^ | 6 | 0.75 | 511.87 | 123.60 |
| Mod# 22 | MDE, RAIN, AET, WI, WI^2^ | 5 | 0.733 | 512.88 | 124.61 |
| Mod# 23 | IFO, IFO^2^, MDE, RAIN, AET, Stemp, WI, WI^2^ | 8 | 0.743 | 519.42 | 131.15 |
| Mod# 24 | IFO, IFO^2^, MDE, RAIN, AET, WI, WI^2^ | 7 | 0.722 | 520.95 | 132.68 |
| Mod# 25 | IFO, IFO^2^, MDE, RAIN, Stemp, WI, WI^2^ | 7 | 0.721 | 521.34 | 133.07 |
| Mod# 26 | MDE, Stemp, WI, WI^2^ | 4 | 0.635 | 528.97 | 140.70 |
| Mod# 27 | MDE, RAIN, Stemp, WI, WI^2^ | 5 | 0.648 | 529.52 | 141.25 |
| Mod# 28 | MDE, RAIN, WI, WI^2^ | 4 | 0.631 | 529.68 | 141.41 |
| Mod# 29 | IFO, IFO^2^, MDE, RAIN, WI, WI^2^ | 6 | 0.656 | 530.86 | 142.59 |
| Mod# 30 | MDE, WI, WI^2^ | 3 | 0.606 | 531.10 | 142.83 |
| Mod# 31 | IFO, IFO^2^, MDE, Stemp, WI, WI^2^ | 6 | 0.637 | 534.08 | 145.81 |
| Mod# 32 | IFO, IFO^2^, MDE, WI, WI^2^ | 5 | 0.595 | 537.90 | 149.63 |
